# A Materia Medica Pura

**Published:** 1890-11

**Authors:** 


### A MATERIA MEDICA PURA.

To those who watch with care the tendency of homœopathic literature, its downward course can scarcely be unnoticed. Of bookmaking we have, indeed, an abundance; of homœopathic books we are sadly lacking. Dr. Lilienthal has alluded to the need of newer and better translations of Hahnemann's *Materia Medica.* Of this there *is* great need. It has always been a misfortune and a serious hindrance to the development of Homœopathy that Hahnemann's works were so poorly translated. It is true, we have a re-translation of the *Materia Medica Pura,* embodied in Allen's *Encyclopædia,* which is to-day the best work on materia medica we possess. But the *Encyclopædia* is by no means faultless; yet, such as it is, we should be thankful for it. In such a large work errors will be found, yet the effect of these errors is in a measure lessened by the repetition of symptoms, which are so numerously given. A symptom repeated over and over again, in different terms, cannot be wrongly stated every time. So, when one reads a symptom in two or three differently worded sentences he can easily get at the meaning of the provers. In the so-called *condensed* books on materia medica an error once stated, remains without chance of correction. The use of these *condensed* works is responsible for many failures in practice. None of them are thoroughly reliable, and none of them give a true picture of the drugs treated. The homœopathic school is yet to produce its *Materia Medica Pura,* with the additions which time has added to Hahnemann's labors. This may seem to be a very sweeping statement but *it is true.* The great victories of Homœopathy in the past have been won by those who studied the *Materia Medica,* not by those who relied upon repertories or *condensed* works. The homœopaths of to-day who make the most brilliant cures rely upon and study the old works upon materia medica. The homœopath of the future will—if the present work of *condensing and copying* goes on—have no reliable materia medica.

One writer copies from another, only to repeat, or, maybe, to increase, the errors of the former. And so goes on the manufacture of homœopathic literature! Of hasty, inconsiderate, and unreliable works our school has seen quite enough. What we need is a faithful and reliable compilation of the old remedies, with such new symptoms as are without the shadow of doubt reliable, and a complete rendering of the reliable symptoms of such new remedies as are well proven. We need no great display of large and elegantly gotten up books; we want only what is true, reliable, and useful. Less than this we cannot do without; more than this is deceptive and misleading. Who will give us a real *Materia Medica Pura*, which shall contain all that is true, both of the new and of old drugs?

The homœopathic school must have such a *Materia Medica Pura*, or it dies! E. J. L.

